# Acute Effect of High-Intensity Interval Training on Postprandial Glycemia in Overweight and Obese Individuals: A Scoping Review

**DOI:** 10.3390/nu17081364

**Published:** 2025-04-17

**Authors:** Hugo Alejandro Carrillo-Arango, David Alejandro Gonzalez, Leidy Tatiana Ordoñez-Mora, Miguel Alejandro Atencio-Osorio, Héctor Reynaldo Triana-Reina, Mikel Izquierdo

**Affiliations:** 1 Grupo de Investigación en Deporte de Rendimiento (GRINDER), Programa de Educación Física y Deporte, Universidad del Valle, Cali 760042, Colombia; hugo.carrillo@correounivalle.edu.co (H.A.C.-A.); david.bustamante@correounivalle.edu.co (D.A.G.); miguel.atencio@correounivalle.edu.co (M.A.A.-O.); 2 Facultad de Salud, Programa de Fisioterapia Grupo de Investigación Salud y Movimiento, Universidad Santiago de Cali, Cali 760035, Colombia; 3 Facultad de Cultura Física, Deporte y Recreación, GICAEDS, Universidad Santo Tomás, Bogotá 110231426, Colombia; hectortriana@usta.edu.co; 4 Navarrabiomed, Hospital Universitario de Navarra (HUN), Universidad Pública de Navarra (UPNA), Instituto de Investigación Sanitaria de Navarra (IdiSNA), 31000 Pamplona, Spain; mikel.izquierdo@gmail.com; 5 CIBER of Frailty and Healthy Aging (CIBERFES), Instituto de Salud Carlos III, 28029 Madrid, Spain

**Keywords:** blood glucose, exercise, glycaemia, postprandial, review, diabetes

## Abstract

Background/Objectives: High-intensity interval training (HIIT) has emerged as an effective strategy for mitigating postprandial glycemia in overweight or obese individuals. This scoping review aims to examine randomized controlled trials (RCTs) conducted between 2008 and 2024 that evaluated the impact of HIIT on acute postprandial glycemic response. Methods: A comprehensive search strategy was employed using terms such as “high-intensity interval training (HIIT)” and “postprandial glycemia”, combined with Boolean operators, with no restrictions on study type. Electronic databases searched included PubMed, SPORTDiscus, Scopus, and Web of Science from their inception through 2024. Of the 67 studies that met the inclusion criteria, five RCTs were selected for final analysis. All selected studies involved individuals with a body mass index (BMI) ≥ 25. Results: Each of the five included RCTs featured at least one HIIT intervention group, with variations in frequency, duration, intensity, and testing protocols. Despite differences in glucose tolerance test timelines, the glucose-loading protocol (75 g) and exercise interventions demonstrated minimal heterogeneity across studies. The findings suggest that short-term HIIT interventions may positively influence acute postprandial glycemic responses in overweight and obese populations. Conclusions: Short-term HIIT appears to be a promising intervention for improving postprandial glycemic control in individuals with elevated BMI. Future research is warranted to further elucidate both the acute and long-term effects of HIIT, particularly the role of skeletal muscle in regulating systemic glucose levels in this population.

## 1. Introduction

Overweight and obesity are escalating public health concerns of global significance, affecting over 1.9 billion adults, with alarming prevalence rates in both developed and developing nations [1]. According to the World Health Organization (WHO), in 2022, 2.5 billion adults over the age of 18 were classified as overweight, with more than 890 million individuals categorized as obese, representing 16% of the global adult population [2,3]. This figure reflects a 100% increase between 1990 and 2022 [4]. The surge in obesity is largely attributed to sedentary lifestyles, dietary shifts, and the rapid pace of urbanization [5]. Moreover, obesity is strongly linked to a heightened risk of chronic conditions such as type II diabetes mellitus (T2D), which is associated with increased insulin resistance (IR) or metabolic syndrome, musculoskeletal disorders, various cancers, and cardiovascular diseases [6,7]. The impact of these conditions places an immense burden on healthcare systems and adversely affects the quality of life of individuals suffering from obesity [8]. The global economic burden of chronic diseases is forecasted to hit $47 trillion by 2030 [9].

Obesity is linked to metabolic alterations, including insulin resistance (IR) and impaired glucose regulation [10]. Excess abdominal adipose tissue releases free fatty acids, which interfere with the action of insulin in both the muscles and liver, leading to reduced glucose uptake and increased hepatic glucose production. Over time, the compensatory response of the pancreas becomes insufficient, contributing to the development of metabolic diseases [11]. Additionally, insulin resistance induces inflammation and oxidative stress, significantly raising the risk of cardiovascular complications and highlighting the need for early intervention [10,12].

In this context, physical exercise (PE) is a vital strategy for the prevention and management of obesity and being overweight, providing numerous benefits for metabolic, cardiovascular, and mental health [13]. Regardless of the type, regular PE can significantly reduce body fat, improve body composition [14], and lower the risk of non-communicable diseases. Furthermore, PE enhances insulin sensitivity, lowers blood pressure, and supports a healthy lipid profile [15]. High-intensity interval training (HIIT) has been proposed as a practical exercise modality for individuals with overweight or obesity, characterized by short, intense exercise sessions interspersed with active recovery periods. Multiple studies have demonstrated its impact on body composition by reducing adipose tissue and improving cardiorespiratory capacity [16,17]. Other research has reported significant improvements in insulin sensitivity and metabolic function [18]. Exercise has been shown to improve insulin sensitivity, lipid profiles, and blood pressure, while contributing to the reduction in central obesity [19]. Both aerobic and resistance training have been effective in reducing cardiometabolic risk, with structured exercise programs demonstrating significant improvements in metabolic health indicators [20] as well as a significant decrease in waist circumference [21].

With respect to energy substrate utilization, lipids serve as a denser energy source and are favored in fasting states or during prolonged exercise, whereas glucose is the primary source during high-energy, short-duration activities like HIIT. The differential utilization of these substrates is regulated by hormonal and enzymatic factors, with insulin playing a central role in promoting postprandial glucose utilization and lipid storage [22]. Despite the promising evidence supporting the role of HIIT in glycemic control, several significant uncertainties persist regarding its precise mechanisms, particularly concerning its acute and long-term effects on skeletal muscle and its contribution to systemic glycemic regulation. These uncertainties remain unresolved.

Therefore, this scoping review aims to summarize the most significant findings on the effects of HIIT in overweight or obese patients, specifically regarding postprandial glycemic responses at various time points. It offers a critical examination of the underlying mechanisms, particularly those occurring in skeletal muscle following exercise.

## 2. Materials and Methods

As our purpose was not to inform clinical practice but to identify evidence on HIIT protocols for controlling postprandial glycemia in overweight and obese individuals, a scoping review, rather than a systematic review, was selected for our methods. This study corresponds to a scoping review [23]. We followed the Preferred Reporting Items for Systematic Reviews and Meta-Analyses Extension for Scoping Reviews [24,25] checklist in reporting this manuscript.

Therefore, the objective was to map, identify, and describe the available evidence in the literature, rather than to evaluate effectiveness or conduct a meta-analysis, which would correspond to a systematic review [26,27].

### 2.1. Search Strategy

Four electronic databases (PubMed, SPORT Discus, Scopus, and Web of Science) were searched for articles published after 2008 until Jul 2024 using a combination of terms to find articles focused on (“obese” OR “obesity” OR “overweight”) AND (“HIT” OR “HIIT” OR “HIFT” OR “circuit-based” OR “aerobic interval training” OR “high-intensity interval training” OR “sprint interval training” OR “High-Intensity Intermittent Exercise” OR “High-Intensity Intermittent”) AND (“glucose tolerance” OR “insulin tolerance test” OR “OGTT” OR “acute response” OR “postprandial period” OR “postprandial periods”). Additionally, we searched for reference lists and personal files of relevant narrative and systematic review articles. No language restrictions were applied.

### 2.2. Article Selection

The articles included met the following criteria: (i) at least one intervention group involved cardiovascular or high interval exercise training; (ii) they included randomized controlled trials (RCTs); (iii) adults (18 years and older) were overweight or obese (BMI ≥ 25); (iv) protocols measuring immediate glycemic/insulin responses using glucose load tolerance tests were adopted; and (v) they included a control group with either another type of PE or no intervention. Articles published in any language were included in the study. Articles were excluded for any of the following reasons: (i) they were pilot works or quasi-experimental, focused solely on glycemic responses prior to food intake, and (ii) involved long-duration HIIT interventions.

### 2.3. Inclusion Criteria

Studies were included if they examined the effects of high-intensity interval training (HIIT) protocols on postprandial glycemia in overweight or obese individuals. Eligible studies had to involve human participants classified as overweight or obese based on standard body mass index (BMI) criteria. Only original, peer-reviewed quantitative studies—including randomized controlled trials and clinical trials—were considered. Additionally, studies had to report at least one outcome related to postprandial blood glucose levels. Publications in English or Spanish, and those published between 2008 and 2024, were eligible for inclusion.

### 2.4. Exclusion Criteria

Studies were excluded if they were narrative reviews, systematic reviews, meta-analyses, editorials, commentaries, opinion pieces, or conference abstracts. Research involving animal models or in vitro experiments was also excluded. Additionally, studies that included participants with comorbidities unrelated to glycemic control (e.g., cancer, renal disease, severe cardiovascular conditions) were not considered. Likewise, studies involving menopausal women, smokers, or individuals who consumed alcohol were excluded due to the potential influence of these factors on metabolic outcomes. Articles without accessible full texts, studies lacking relevant outcome data on postprandial glycemia, or those not published in English or Spanish were also excluded.

### 2.5. Data Abstraction

Data abstraction was performed independently and in duplicate by three trained researchers (HCA, DAGB, and LTOM). Reviewers screened the titles, abstracts, and full texts of all retrieved studies. Disagreements were resolved through discussion, and when necessary, conflicts between reviewers were resolved by consensus or, if unresolved, in consultation with a senior researcher (MI). To assess inter-rater reliability during the selection process, the intraclass correlation coefficient (ICC) was calculated for a random sample of 20% of the studies. The ICC was 0.86 (95% CI: 0.80–0.92), indicating good agreement between reviewers.

The study selection process began with the calibration of the study criteria. Article selection was facilitated through the Rayyan virtual tool [28] (https://rayyan.qcri.org/welcome, accessed on 5 June 2024). At this stage, duplicate articles were identified and removed. Titles and abstracts were reviewed, and those that met the inclusion criteria were filtered. Data extraction was independently conducted using an Excel format, which included: first author, year of publication, study design, country, sample size, age, study type, exercise protocol used, comparison protocol, and reported changes in glycemic control (glucose and/or insulin levels). The results are presented as means and standard deviations for each reported outcome, and all authors verified the accuracy of the information.

### 2.6. Risk of Bias

The risk-of-bias methodology of the included studies was assessed using the PEDro scale [29], an 11-item checklist with a maximum score of 10. This instrument evaluates key criteria such as random allocation, allocation concealment, baseline comparability of characteristics, blinding of participants, therapists, and assessors, availability of outcome data for at least 85% of participants for at least one primary outcome, intention-to-treat analysis, statistical comparisons between groups, and their respective outcomes.

### 2.7. TIDieR Checklist and Reporting Integrity

Additionally, the quality of the replication of the interventions was assessed using the TIDieR tool [30], which comprises 12 key elements. These include the name of the intervention; the theoretical framework supporting the exercise-based intervention; the materials and procedures used; and the frequency, duration, and modifications applied to ensure reproducibility. Investigators (HCA, DAGB, LTOM) independently applied the TIDieR checklist to the included RCTs. As recommended by the TIDieR committee, the checklist was completed following the TIDieR guide [30], which provides an explanation and elaboration for each element. Each item is scored as Yes or No. Only items that were clearly fulfilled were rated Yes, and partially fulfilled items were rated No. Three investigators discussed any disagreements, and unresolved cases were settled by a fourth investigator. Finally, for each item, the percentage of interventions in the included studies that reported on the item was calculated.

## 3. Results

**Figure 1:**
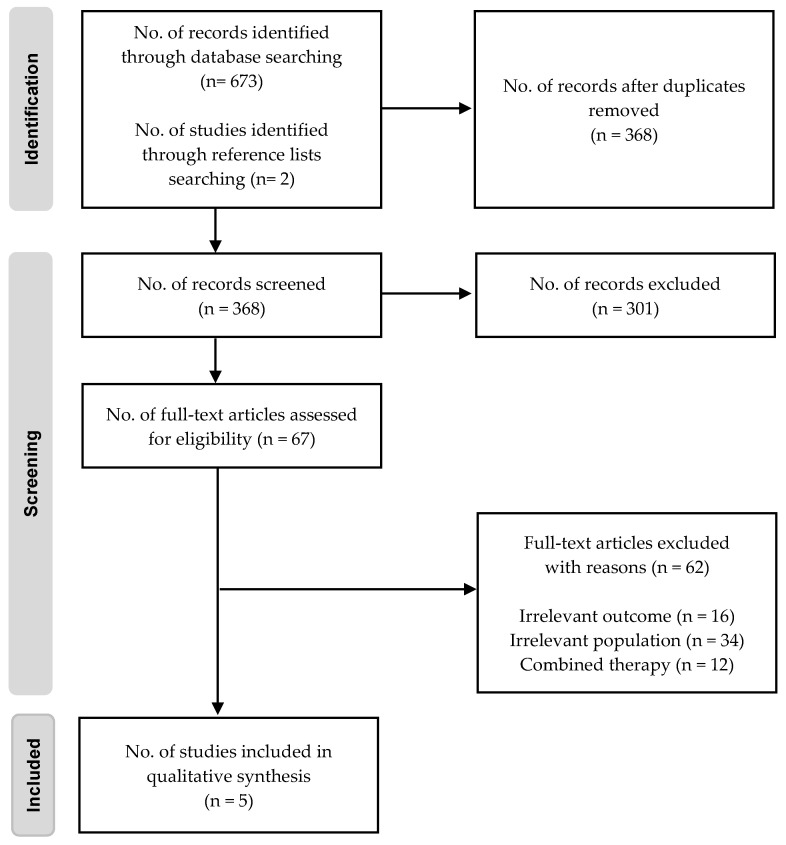
Flow diagram of included and excluded studies.

The article searches across databases yielded 675 studies. After exporting to Rayyan and removing duplicates, 368 articles remained for title and abstract reviews. Following this selection, the eligibility of the 67 articles was evaluated by reviewing the full text. Finally, five RCTs met all selection criteria and were included in the analysis [31,32,33,34,35], as shown in Figure 1.

### 3.1. Study Characteristics

The selected studies were RCTs conducted over different durations: three days [32], five days [31], one week [35], and two weeks [33,34], allowing for the comparison of results across trials. None of the studies included follow-up periods extending beyond 25 h after the interventions were concluded. These studies were conducted in various countries, including Australia [32], Colombia [31], China [35], Finland [34], and the United Kingdom [33], all of which were originally published in English. A total of 122 participants were involved, of whom 71% were male, 7% female, and 22% were unreported. The age range of the participants was 18–55 years.

In all studies, the participants acted as both control and intervention groups. Interventions were performed using cycle ergometers or bicycles. All studies [31,32,33,34,35] compared the effects of HIIT in the absence of PE. Additionally, two studies assessed postprandial glucose levels after high-intensity exercise, with recovery periods extending beyond 24 h, while also examining the glycemic response in the absence of PE. These studies reported that patients had no restrictions regarding physical exercise and were classified as overweight or obese.

### 3.2. Main Findings

Regarding the number of publications per year, there has been a progressive increase in scientific output related to HIIT protocols for controlling postprandial glycemia in overweight and obese individuals. Most of the studies were published after 2018 [31,32,34], with the year 2012 also standing out due to two publications on the same topic [33,35]. In contrast, since 2018, there has been a notable growth in publications, suggesting a rising interest in this topic in recent years.

The primary outcome of this review was the demonstrated positive effects of HIIT exercise interventions in mitigating postprandial glycemic response in overweight and obese adults. All studies evaluated glycemic response using the oral glucose tolerance test (OGTT), a widely accepted method for evaluating how the body processes glucose, and a reliable diagnostic tool for diabetes mellitus and prediabetes [36]. Typically, during the OGTT, participants consumed a glucose solution (commonly 75 g diluted in 250–300 mL of water), and blood glucose levels were measured at regular intervals (e.g., every 30 min over a two-hour period) to observe how the body metabolizes glucose load [37]. While many studies adhered to this traditional format, some modified the post-ingestion sampling intervals and timing of interventions (15, 30, 60, 90, 120 min; 25 and 96 h) to assess responses in both the experimental and control groups.

All trials evaluating high-intensity physical exercise employed a stationary bicycle, with the most used protocol being Sprint Interval Training (SIT) or its variations. SIT involves performing short sprints at maximal effort, followed by periods of rest or low-intensity activity. Variations in sprint duration (ranging from 10 to 30 s) and rest intervals are crucial, as they allow for adjustments in workload and improve metabolic adaptation [38]. In this review, the sprints ranged from 30 to 60 s [31,32,33,34,35], with active recovery typically set at 50% to 60% of maximal intensity. These variations are designed to optimize both cardiovascular and metabolic responses, providing improvements in both anaerobic and aerobic capacity in a shorter time frame than with continuous lower-intensity exercise [39]. Three of the studies [31,32,33,34,35] focused solely on the SIT intervention without comparing it to other training modalities. In contrast, two studies [32,34] compared SIT to lower-intensity, longer-duration exercise (30, 40, and 60 min) at 60% intensity.

SIT has been shown to significantly reduce blood glucose levels, both at rest and in response to food intake, specifically postprandial glycemia [40]. This type of training was effective in mitigating postprandial glucose levels at 15, 30, 60, 90, and 120 min post-ingestion when compared to control groups in overweight and obese individuals. However, the long-term effects of SIT on glycemic control remain controversial. One study reported no significant reduction in postprandial glucose levels after 25 h, while another demonstrated significant differences following two weeks of training, with sustained effects observed up to 96 h post-exercise. These findings suggest that multiple sessions of SIT exercise may yield more pronounced long-term mitigation, facilitating glucose uptake by muscles and contributing to better glycemic control after meals [41]. Although some studies did not assess insulin sensitivity, when compared to lower-intensity exercise of a longer duration (120 s) at less than 60% intensity, sensitivity was not significantly higher [35], nor was it significantly different from the control groups, Table 1.

### 3.3. Intervention Description

The protocols used consisted of high-intensity exercise with variations, including eight repetitions of 30 s at maximum intensity on a cycle ergometer, with a resistance of 0.075 kg (Wingate test) [31]. Another study employed an incremental test on a cycle ergometer, increasing by 25 W every 2 min until exhaustion [32]. Comparable protocols included four sprints of 30 s with 4.5 min of recovery [33,34,35], and another study maintained a maximum sprint duration of 200 s [33].

### 3.4. Description of the OGTT Protocol Used

The glucose load used in the studies contained 75 g of glucose, following the standard protocols for oral glucose tolerance tests. Measurements included pre-glucose load values at intervals of 30 min [31,33], 1 h post-exercise [32,33,34], 1.5 h [31,33], 3.5 h [31], 4.5 h [31], 5.5 h [31], three days later, and two weeks later [34].

### 3.5. Results of Risk of Bias

Although quality assessment is not a mandatory component of scoping reviews, the PEDro scale was applied as an additional tool to describe the general methodological quality of the studies. This approach aimed at providing a broader understanding of the strength of the available evidence, without excluding studies based on their score, many of the included studies (3 of 5) were assessed as being at low risk of bias, defined as ≥6 on the PEDro scale (Table 2). All trials included were of parallel, randomized design.

### 3.6. Overall Reporting

The integrity of the reports varied from 0% to 100% across TIDieR items (Table 3). The brief name, materials, procedures, expertise, setting, timing, and rationale for the intervention were the most consistently reported items across all interventions (five items, 100%), followed by the study justification and the extent of planning (five items, 80%). In contrast, the provider, modifications, and tailoring were not reported at all (5 items, 0%). Half of the TIDieR items were reported in all interventions, with two items reported in 80% of the interventions (items 2 and 11), and only one item was reported in 60% (item 12). Additionally, five of the seven items (3, 4, 6, 7, 8) deemed essential for replication [30] exhibited 100% reporting.

## 4. Discussion

This review highlights a growing scientific interest in the application of HIIT protocols to manage postprandial glycemia in overweight and obese individuals, particularly after 2018. Research in this area appears to be gaining momentum, with a focus on optimizing protocol variables such as intensity, session timing relative to meals, and frequency. Furthermore, recent studies increasingly explore specific metabolic outcomes such as glucose variability, insulin sensitivity, and glycemic excursions, indicating a broadening of the research scope. These trends suggest the need for continued investigation and knowledge translation to clinical and public health settings.

The predominant variable assessed across studies was postprandial glucose, with a strong emphasis on evaluating different exercise intensities using cycle ergometers. Four studies compared postprandial glucose responses after HIIT versus no-exercise conditions [31,33,34,35]. Most of the protocols (n = 4) used cycling-based HIIT sessions with 30-s intervals [31,33,34,35]. The timing of exercise relative to meals varied significantly across studies, ranging from 15 to 120 min post-meal [31,32,33,35], and even up to 8, 25, or 96 h, or two weeks later [32,34]. All studies employed a standardized 75-g oral glucose tolerance test (OGTT) to assess glycemic response. The main physiological outcomes measured were glucose and insulin levels. Participant demographics showed a predominance of male subjects (n = 77), followed by studies that did not report sex (n = 26), and a notably lower representation of female participants (n = 9). This highlights a significant gender gap in the existing literature and underscores the need for further research focusing on women. All participants were adults aged over 18 but under 50 years.

The beneficial effects of PA, including structured exercise, are well documented. This study evaluated the acute effects of HIIT programs in overweight and obese sedentary individuals. The findings corroborate improvements in post-exercise blood glucose concentrations at 30, 60, 90, and 120 min, measured at various intervals following glucose ingestion, including up to 25 h post-exercise. This exercise modality can reduce glucose and insulin levels during postprandial periods in healthy adults, suggesting a critical role in enhancing cardiometabolic health. However, evidence regarding the optimal intensity, type, and duration of exercise for reducing glycemic markers remains inconsistent; thus, it is essential to evaluate the underlying mechanisms of a single session of HIIT and its effects on postprandial glucose.

Previous findings indicate that an acute session of HIIT induces substantial improvements in postprandial glucose levels on the day of exercise. In this scoping review, the included studies consistently demonstrated that HIIT significantly reduced postprandial blood glucose in overweight and obese individuals. Most studies have reported better acute glycemic control following a session of HIIT than following rest or continuous moderate-intensity exercise. However, some studies found modest or variable effects, which may be attributed to differences in HIIT protocols, food intake, participant demographics, degree of adiposity, exercise intensity, population characteristics, comorbidities, and exercise type.

Improvements in postprandial glycemia may include an increase in glucose uptake in skeletal muscles owing to enhanced blood flow and the activation of exercise-related metabolic pathways. HIIT may also induce changes in the expression of proteins involved in glucose metabolism, such as glucose transporter type 4 (GLUT4), which facilitates the entry of glucose into muscle cells. Many of the included studies were randomized controlled trials, which strengthens the validity of the findings. However, the variability in the HIIT protocols and participant characteristics limits the generalizability of the results.

## 5. Conclusions

This scoping review provides an overview of the existing evidence on the effects of high-intensity interval training (HIIT) on postprandial glycemia in overweight and obese individuals. The findings suggest that HIIT protocols using cycle ergometers—typically involving 30-s intervals at high intensity—are frequently employed and show beneficial effects on glycemic control when performed within 15 to 120 min after meal ingestion. Based on the identified studies, clinicians and exercise professionals may consider prescribing HIIT sessions with short intervals (30 s of intense effort) interspersed with recovery periods, with total durations ranging from 10 to 20 min. A 75 g oral glucose tolerance test (OGTT) was consistently used for assessment, and future studies should adopt standardized evaluation tools to allow for better comparisons across trials. Furthermore, given the low representation of female participants, future research should prioritize sex-specific analyses. Overall, HIIT appears to be a promising, time-efficient strategy for managing postprandial glycemia in this population, but personalized protocols are essential to maximize safety and effectiveness.

From a practical standpoint, the findings of this review suggest that HIIT may serve as a viable and effective strategy for managing postprandial glycemia in overweight and obese individuals. This is particularly relevant in contexts where lifestyle interventions are crucial for the prevention and treatment of metabolic diseases. Current evidence suggests that short-duration HIIT may be an effective alternative to conventional exercise regimens for improving insulin sensitivity and glycemic control in individuals with obesity. However, the studies also highlight certain limitations, such as small sample sizes and the need for further research to evaluate effects in specific populations, such as prediabetic individuals. Overall, these findings support the potential of brief, intense exercise as a viable strategy for enhancing metabolic health in overweight or obese adults.

Finally, it is important to emphasize that only five randomized controlled trials met the inclusion criteria, thereby limiting the overall strength and generalizability of the conclusions. While this scoping review offers a valuable preliminary synthesis of the available literature, the limited number of high-quality RCTs highlights a significant gap in the current evidence base. Consequently, further rigorously designed and adequately powered trials are essential to support more definitive and evidence-based recommendations. The scarcity of eligible studies also underscores the urgent need for additional research specifically examining the effects of high-intensity interval training on postprandial glycemia in overweight and obese populations.

### Future Directions and Limitations

Future studies should aim to include more diverse populations, particularly females, to address the current sex imbalance in the literature. Standardized HIIT protocols and consistent timing in relation to meals would facilitate more robust comparisons across studies. Additionally, the use of continuous glucose monitoring could provide more detailed insights into glycemic responses beyond the traditional OGTT. From a practical standpoint, HIIT interventions should be adapted to individual fitness levels and metabolic conditions to ensure both safety and efficacy. Despite the promising outcomes, several limitations were identified among the included studies, including small sample sizes, short intervention durations, lack of long-term follow-up, and insufficient reporting on adherence and adverse events. Addressing these methodological gaps in future trials will enhance the reliability and applicability of findings to real-world clinical and community settings.

## Figures and Tables

**Table 1:** Characteristics and main results of the studies.

Author and Year	Sample Size	Sex	Average Age	BMI	Intervention Group: Type (T), Duration (D), Repetitions (R), and Frequency (F)	Comparison	OGTT Protocol	Main Results
Carrillo-Arango et al., 2023 [31]	n = 33	H = 24 M = 9	33 ± 8	29 ± 4	T = Cycling (Wingate 0.075 kg * BW kg) D = 30 s R = 8 times F = One session	No exercise (same control group)	After a 10 to 12-h fast, participants ingested an oral solution of 75 g of glucose dissolved in 250 mL of water. The response was measured at 60, 90, and 120 min following the intervention.	Glucose after 60 min was lower in the SIT group (88.26 ± 3.74) compared to the resting group (97.80 ± 3.57).
Honkala et al., 2020 [34]	n = 53 GC = 28 GI = 26	H = 28 NR = 26	49 ± 8	26.1 ± 2.4	T = Cycling (SIT) (GC = 7.5% * BW) (GI = 10% * LBM kg) D = 30 s 4 min recovery R = Initial (4) up to (6) F = Two weeks	T = Cycling (MICT 60% of VO2 peak) D = Initial (40 min) up to (60 min) R = One session F = Two weeks	Participants ingested an oral solution of 75 g of glucose dissolved in 250 mL of water. The response was measured at baseline and 96 h later, and this was repeated after 2 weeks.	GC = *F − Glu* (mmol/L) Pre 5.44 (5.25–5.64); Post 5.74 (5.52–5.96). *HbA1c* (mmol/L) Pre 36.93 (35.19–38.66); Post 34.72 (32.94–36.50).HbA1c (%) Pre 5.5 (5.4–5.7); Post 5.3 (5.2–5.5). GI = *F − Glu* (mmol/L) Pre 7.20 (6.86–7.56); Post 7.16 (6.80–7.55). HbA1c (mmol/L) Pre 39.64 (37.33–41.96); Post 37.65 (35.27–40.02). HbA1c (%) Pre 5.8 (5.6–6.0); Post 5.6 (5.5–5.8)
Nie et al., 2012 [35]	n = 10	H = 10	25.3 ± 5.1	32.0 ± 4.0	T = Cycling (HIE 5% BW) D = 30 s 4 min (active recovery) R = 4 sprints F = One session	T1 = Recovery (REC) D = 24 h after HIE T2 = No exercise (CON) D = 7 days after HIE	After the exercise intervention (HIE) or no exercise (REC and CON), subjects underwent an OGTT by ingesting 75 g of anhydrous glucose dissolved in 300 mL of water. Blood glucose levels in each test were measured before and at 30, 60, 90, and 120 min after glucose ingestion (IG).	Glucose levels at 30 min post-GI were lower in the HIE trial (6.9 ± 0.4 mmol/L) compared to CON (9.0 ± 0.4 mmol/L) and REC (8.8 ± 0.4 mmol/L) (*p* < 0.05). At 120 min, glucose in REC (5.7 ± 0.3 mmol/L) was lower than in CON (6.9 ± 0.4 mmol/L) (*p* < 0.05). The AUC of glucose in CON (890 ± 43 mmol/L/min) was higher than in HIE (834 ± 40 mmol/L/min) and REC (846 ± 32 mmol/L/min) (*p* < 0.05), with no differences between HIE and REC (*p* > 0.05).
Raman et al., 2018 [32]	n = 15	H = 15	From 18 to 44	29.0 ± 3.1	T = Cycling (HIIE 100% VO2 max) D = 1 min 4 min (active recovery 50%) R = 6 times F = One session (30 min)	T1 = Cycling (CME 60% VO2 max) D = 30 min R = 1 time F = One session (30 min) T2 = No exercise (Baseline)	Participants, after an 8 h fast, underwent an oral glucose tolerance test (75 g). Capillary blood samples were taken before and at 15, 30, 60, 90, 120 min, and 25 h post-ingestion to measure glucose	The plasma glucose AUC was significantly lower on Day 2 (after exercise) (mean difference: −0.36 mmol/L; 95% CI: [−0.65, −0.08); ES: 0.755) and 25 h later (mean difference: −0.56 mmol/L; 95% CI: [−0.79, −0.34); ES: 1.078). Exercise reduced glucose concentrations at 60, 90, and 120 min of the OGTT on Day 2, with a 5.2 mmol (93.68 mmol) decrease (4.4%; *p* = 0.003) in the glucose AUC compared to the pre-exercise AUC; 60 and 120 min after the OGTT.
Whyte et al., 2013 [33]	n = 10	H = 10	From 18 to 40	26.9 ± 6.2	T1 = Cycling (SIT 0.065 * LBM kg) D = 30 s 4 min (active recovery 50%) R = 4 times F = 30 s T2 = Extended sprint (<50 rpm) R = 1 time F = 200 s	T = No exercise	After an overnight fast (12 h), subjects consumed a drink containing 82.5 g of glucose monohydrate (equivalent to 75 g of anhydrous glucose) in 300 mL of water, and blood samples were then taken at 30-min intervals for 120 min	The ISI was 44.6% higher after ES than after CON (9.4 ± 2.1 vs. 6.5 ± 1.3; *p* = 0.022), but it did not differ significantly between SIT and CON (6.6 ± 0.9 vs. 6.5 ± 1.3; *p* = 0.208). Glucose results: (SIT: 6.6 ± 0.9 mmol/L), (Extended Sprint (ES): 9.4 ± 2.1 mmol/L), and (Control (CON): 6.5 ± 1.3 mmol/L). POST (SIT: 5.34 ± 0.16 mmol/L), (Extended Sprint (ES): 5.32 ± 0.10 mmol/L), (Control Group (CON): 5.43 ± 0.15 mmol/L)

Legend: n: Participants number. H: Males. F: Females. SIT: Sprint interval training. GC: Control group. GI: Intervention group. NR = Not Reported. MICT: Moderate-intensity continuous training. HIE: High-intensity exercise. BW: Body weight. BW kg: Body weight in kilograms. LBM kg: Lean body mass in kilograms. * multiplication.

**Table 2:** Risk of bias among included trials.

Study	Items											Total
	1	2	3	4	5	6	7	8	9	10	11	
Carrillo-Arango et al., 2023 [31]	✓	✓	X	✓	X	X	X	✓	✓	✓	✓	6
Honkala et al., 2020 [34]	✓	✓	X	X	X	X	X	✓	✓	✓	✓	5
Raman et al., 2018 [32]	✓	X	X	✓	✓	X	X	✓	✓	✓	✓	6
Nie et al., 2012 [35]	✓	X	X	✓	X	X	X	✓	✓	✓	✓	5
Whyte et al., 2013 [33]	✓	✓	X	✓	X	X	X	✓	✓	✓	✓	6

Legend: Items: 1: eligibility criteria; 2: random assignment; 3: hidden allocation; 4: hidden allocation; 5: initial comparability; 6: blind dealers; 7: blind evaluators; 8: proper follow-up; 9: intention-to-treat analysis; 10: cross-group comparisons; 11: point estimates and variability; the eligibility criteria do not contribute to the overall score.

**Table 3:** TIDieR checklist and reporting integrity.

TIDieR Items	RCTs (n)
1. Brief name	5 (100%)
Provide the name or a phrase that describes the intervention	All
2. Why	4 (80%)
Describe any rationale, theory, or goal of the elements to the intervention	H, N, R, W
3. Materials	5 (100%)
Describe any physical or informational materials used in the intervention	All
4. Procedures	5 (100%)
Describe each of the procedures, activities and process	All
5. Who provided (Background)	0 (0%)
Describe the disciplinary background of the provider	None
6. Who provided (Expertise)	5 (100%)
Describe the expertise, experience, or specific training of the provider	All
7. Where	5 (100%)
Describe the type(s) of location(s) where the intervention occurred	All
8. Where and how	5 (100%)
Describe number of sessions, the duration and the intesity of the intervention.	All
9. Tailoring	0 (0%)
Describe what, why, when and how the intervention was adapted or tritated.	None
10. Modifications	0 (0%)
Describe the changes if the intervention was modified	None
11. How well (Planned)	4 (80%)
Describe how, whom and if any strategies were used to maintain adherence.	C, N, R, W
12. How well (actual)	3 (60%)
Describe the extent to which the intervention was delivered as planned.	C, N, R

Legend: C: Carrillo-Arango et al., 2023 [31]; H: Honkala et al., 2020 [34]; N: Nie et al., 2012 [35]; R: Raman et al., 2018 [32]; W: Whyte et al., 2013 [33].

## Data Availability

Secondary data were used and will be made available by the authors upon request, subject to privacy-related restrictions on the analyzed documents.
